# Supplementary material for: Proofing Direct-Seeded Rice with Better Root Plasticity and Architecture
Source: Int J Mol Sci. 2021 Jun 4;22(11):6058. doi: 10.3390/ijms22116058 (PMC8199995; doi:10.3390/ijms22116058)
Supplement: Supplementary file 1 [file ijms-22-06058-s001.zip › ijms-1216197-supplementary.pdf]

Table S1 Root traits associated QTLs in direct-seeded rice (DSR) conditions

Trait	Donor	Population	QTLs	Chromosome	Flanking marker	References
Root length	-	HKR47 × MAS26	$qRL_{8.1}$	8	RM8243	[1]
	-	HKR47 × MAS26	$qRL_{9.1}$	9	RM524-RM257 (13.2-66.1 cM)	
	IR94225-B-82-B, IR94226-B-177-B	MASARB25 × PB1460 HKR47 × MAS26	$qRL_{8.2}$	8	RM547-RM331 (58.1-65.0 cM), RM310-RM547 (57.0-58.1 cM)	[1,2]
Root hair length	IR94225-B-82-B, IR94226-B-177-B	-	$qRHL_{1.1}$	1	-	[3]
Root hair density	IR94225-B-82-B, IR94226-B-177-B	-	$qRHD_{1.1}$	1	-	[3]
			$qRHD_{5.1}$	5		
Nodal root number	IR94225-B-82-B, IR94226-B-177-B	-	$qNR_{5.1}$	5	-	[3]
			$qNR_{4.1}$	4		
Root volume	-	-	$qRV_{2.1}$	2	RM485-RM7033 (0.0-7.9 cM)	[4,5]
Root thickness	-	HKR47 × MAS26	$qRT_{1.1}$	1	RM488-RM237 (101.4-112.9 cM)	[4–6]
Fresh root weight	-	MASARB25 × PB1460	$qFRW_{2.1}$	2	RM485-RM7033 (0.0-7.9 cM)	[4,5]
Anaerobic germination	IR93312-30- 101-20-13-66-6	Donor:Khao Hlan On, Recipient: IR64	$qAG_{9.1}$	9	RM8303-RM5526	[7]
			qAG9.2	9		
Early uniform emergence	IR91648-B- 32-B	Donor: Moroberekan Recepeint: Swarna	$qEUE_{11.1}$	11	id1100085-id11001535	[8]
			qEMM1.1	1		[8]
Early vigor	IR94226-B- 177-B	Aus276 × MTU1010	$qEVV_{9.1}$ *(Donor: Aus276)*	9	Flanking markers: id5000759-id5001182, ud9000737-id9002704,id1005271-id1006691; peak markers: id5001182, ud9000737, id1005271	[3]
Drought tolerance	IR74371-46- 1-1	IR74371-46-1-1/2*Sabitri	$qDTY_{12.1}$	12	$qDTY1_{2.1}$ RM28166-RM28199	[4,5,9,10]
			$qDTY_{2.3}$	2	$qDTY_{2.3}$ RM3212-RM250	
			$qDTY_{3.2}$	3	$qDTY_{3.2}$ RM22-RM545	

	IR6322-34- 223	Donors: N22, Apo N22/Swarna, Apo/2*Swarna Ldg Rm8213	$qDTY_{3.1}$	3	$qDTY_{3.1}$: RM416	[6,8,10–12]
			$qDTY_{1.1}$	1	$qDTY_{1.1}$: RM11943-RM12091	
			$qDTY_{2.1}$	2	*qDTY*: RM324	
Lodging resistance	IR91648-B- 289-B	Donor: Swarna	$qLDG_{3.1}$	3	id3000090-id300057	[8]
Lodging resistance	IR91648-B- 289-B	Donor Moroberekan	$qLDG_{4.1}$	4	id4004461-id4009390	[8]

Table S2 Chromosome wise QTLs/genes related to root developmental traits in rice along with their locus ID and encoded protein

Root Phenotype	Gene	Chromosome	Locus ID		Encoded protein	References
			RAP	MSU		
**Crown root**	*OsRAA1*	1	Os01g0257300	LOC_Os01g15340	GTP-binding protein	[13]
	OsYUC1	1	Os01g0645400	LOC_Os01g45760	Flavin monooxygenase	[14]
	OsPIN3t (*OsPIN10a*)	1	Os01g0643300	LOC_Os01g45550	Auxin efflux carrier	[15]
	OsmiR393a	1	-	-	MicroRNA	[16]
	FIB/OsTAA1	1	Os01g0169800	LOC_Os01g07500	Aminotransferase	[17]
	ERF3	1	Os01g0797600	LOC_Os01g58420	AP2/ERF transcription factor	[18]
	REH1/ OsPIN1b	2	Os02g0743400	LOC_Os02g50960	Auxin efflux carrier	[19]
	OsRR2	2	Os02g0557800	LOC_Os02g35180	Type-A response regulator	[20]
	OsCAND1	2	Os02g0167700	LOC_Os02g07120	SCF^{TIR1} ubiquitin ligase	[21]
	CRL1/ARL1	3	Os03g0149100	LOC_Os03g05510	LOB/ASL transcription factor	[22,23]
	OsCOW1 (*OsYUC8*)	3	Os03g0162000	LOC_Os03g06654	Flavin monooxygenase	[24]
	CRL4/ OsGNOM1	3	Os03g0666100	LOC_Os03g46330	Arf-GEF	[25,26]
	OsRR1	4	Os04g0442300	LOC_Os04g36070	Type-A response regulator	[27]
	OsmiR393b	4	-	-	MicroRNA	[16]
	OsMT2b	5	Os05g0111300	LOC_Os05g02070	Metallothionein	[28]
	OsPIN2	6	Os06g0660200	LOC_Os06g44970	Auxin efflux carrier	[29]
	WOX11	7	Os07g0684900	LOC_Os07g48560	WUS-related homeobox protein	[20]
	CRL5	7	Os07g0124700	LOC_Os07g03250	AP2/ERF transcription factor	[27]
	CRL6	7	Os07g0497100	LOC_Os07g31450	CHD family protein	[30]
	OsIAA3 (*OsIAA31*)	12	Os12g0601400	LOC_Os12g40900	Aux/IAA protein	[31]
	OsPID	12	Os12g0614600	LOC_Os12g42020	Serine/threonine kinase	[32]
**Lateral root**	*OsAUX1*	1	Os01g0856500	LOC_Os01g63770	Auxin influx carrier	[33]
	LRL2/ OsCYP2	2	Os02g0121300	LOC_Os02g02890	Cyclophilin	[34]
	OsIAA11	3	Os03g0633500	LOC_Os03g43400	Aux/IAA protein	[35]
	OsIAA13	3	Os03g0742900	LOC_Os03g53150	Aux/IAA protein	[36]
	OsHO1	6	Os06g0603000	LOC_Os06g40080	Heme oxygenase	[29]
	OsORC3	10	Os10g0402200	LOC_Os10g26280	Origin recognition complex protein	[37]

	OsWOX3A/ OsNS (*NAL2*)	11	Os11g0102100	LOC_Os11g01130	WUS-related homeobox protein	[38]
	OsWOX3A/ OsNS (*NAL3*)	12	Os12g0101600	LOC_Os12g01120	WUS-related homeobox protein	[38]
	SOR1	4	Os04g0101800	LOC_Os04g01160	E3 ubiquitin ligase	[39]
**Root hair number**	*OsFH1*	1	Os01g0897700	LOC_Os01g67240	Multidomain protein	[40]
	SRH2	3	Os03g0300000	LOC_Os03g18820	Xyloglucan 6-xylosytransferase	[41]
	OsRHL1	6	Os06g0184000	LOC_Os06g08500	bHLH transcription factor	[42]
	OsEXPA17	6	Os06g0108600	LOC_Os06g01920	Expansin	[43]
	RTH1/ OsAPY1	7	Os07g0682800	LOC_Os07g48430	ATP diphosphohydrolase	[44]
	OsSNDP1	10	Os10g0122600	LOC_Os10g03400	Phosphatidylinositol transfer protein	[45]
	OsEXPA30	10	Os10g0535900	LOC_Os10g39110	Expansin	[43]
	RTH2/ OsCSLD1	10	Os10g0578200	LOC_Os10g42750	Cellulose synthase-like protein	[46,47])
**Root meristem formation**	*QHB*	1	Os01g0854500	LOC_Os01g63510	WUSCHEL-type homeobox protein	[48]
	DOCS1	2	Os02g0236100	LOC_Os02g14120	LRR-RLK	[49]
	OsIAA23	6	Os06g0597000	LOC_Os06g39590	Aux/IAA protein	[50]
	OsSHR1	7	Os07g0586900	LOC_Os07g39820	GRAS transcription factor	[48,51]
	OsSCR1	11	Os11g0124300	LOC_Os11g03110	GRAS transcription factor	[51,52]
**Root length**	*OsSPR1*	1	Os01g0898300	LOC_Os01g67290	Armadillo-like repeat domain protein	[53]
	OsEXPA8	1	Os01g0248900	LOC_Os01g14650	Expansin	[54]
	OsMOGS	1	Os01g0921200	LOC_Os01g69210	Mannosyl-oligosaccharide glucosidase	[55]
	OsCKI1	2	Os02g0622100	LOC_Os02g40860	Putative casein kinase I	[45]
	OsCYT-INV1	2	Os02g0550600	LOC_Os02g34560	Alkaline/neutral invertase	[56]
	OsARF12	4	Os04g0671900	LOC_Os04g57610	Auxin response factor	[57]
	RT/OsGLU3	4	Os04g0497200	LOC_Os04g41970	Endo-1,4-β-D-glucanase	[58,59]
	OsRPK1	5	Os05g0486100	LOC_Os05g40770	LRR-RLK	[60]
	OsDGL1	7	Os07g0209000	LOC_Os07g10830	Dolichyl-diphosphooligosaccharide–protein glycosyltransferase	[61]

	OsGNA1	9	Os09g0488000	LOC_Os09g31310	Glucosamine-6-P acetyltransferase	[62]
	DRO1	9	Os09g0439800	LOC_Os09g26840		[63]
**Nutrient uptake related genes**	*PSTOL1*	12	Os12g0448900	LOC_Os12g26290	receptor-like cytoplasmic kinase	
	OsPT1	3	Os03g0150600	LOC_Os03g05620	inorganic phosphate transporter	[64]
	OsPT8	10	Os10g0444700	LOC_Os10g30790	inorganic phosphate transporter	[53]
	OsNAR2.2	4	Os04g0480200	LOC_Os04g40410	high affinity nitrate transporter	[65]
	OsEXP3	5	Os05g0276500	LOC_Os05g19570	Alpha-expansin-3	[66]
	OsZIP4	8	Os08g0207500	LOC_Os08g10630	Zrt-Irt-like protein 4,metal cation transporter	[67]
	TOND1	12	Os12g0630100	LOC_Os12g43440	thaumatin	[68]

References

[1] Y. Qu, P. Mu, H. Zhang, C.Y. Chen, Y. Gao, Y. Tian, F. Wen, Z. Li, Mapping QTLs of root morphological traits at different growth stages in rice, Genetica. 133 (2008) 187–200. https://doi.org/10.1007/s10709-007-9199-5.

[2] N. Sandhu, A. Singh, S. Dixit, M.T. Sta Cruz, P.C. Maturan, R.K. Jain, A. Kumar, Identification and mapping of stable QTL with main and epistasis effect on rice grain yield under upland drought stress, BMC Genet. (2014). https://doi.org/10.1186/1471-2156-15-63.

[3] N. Sandhu, R.O. Torres, M.T. Sta Cruz, P.C. Maturan, R. Jain, A. Kumar, A. Henry, Traits and QTLs for development of dry direct-seeded rainfed rice varieties, J. Exp. Bot. (2015). https://doi.org/10.1093/jxb/eru413.

[4] J. Bernier, A. Kumar, V. Ramaiah, D. Spaner, G. Atlin, A large-effect QTL for grain yield under reproductive-stage drought stress in upland rice, Crop Sci. 47 (2007) 507–516.

[5] R. Venuprasad, M.E. Bool, C.O. Dalid, J. Bernier, A. Kumar, G.N. Atlin, Genetic loci responding to two cycles of divergent selection for grain yield under drought stress in a rice breeding population, Euphytica. 167 (2009) 261–269. https://doi.org/10.1007/s10681-009-9898-3.

[6] K.H. Ghimire, L.A. Quiatchon, P. Vikram, B.P.M. Swamy, S. Dixit, H. Ahmed, J.E. Hernandez, T.H. Borromeo, A. Kumar, Identification and mapping of a QTL (qDTY1. 1) with a consistent effect on grain yield under drought, F. Crop. Res. 131 (2012) 88–96.

[7] S.A. Angaji, E.M. Septiningsih, D.J. Mackill, A.M. Ismail, QTLs associated with tolerance of flooding during germination in rice (Oryza sativa L.), Euphytica. 172 (2010) 159–168. https://doi.org/10.1007/s10681-009-0014-5.

[8] S. Dixit, A. Grondin, C.R. Lee, A. Henry, T.M. Olds, A. Kumar, Understanding rice adaptation to varying agro-ecosystems: Trait interactions and quantitative trait loci, BMC Genet. (2015). https://doi.org/10.1186/s12863-015-0249-1.

[9] K.K. Mishra, P. Vikram, R.B. Yadaw, B.P.M. Swamy, S. Dixit, M.T.S. Cruz, P. Maturan, S. Marker, A. Kumar, QDTY12.1: A locus with a consistent effect on grain yield under drought in rice, BMC Genet. 14 (2013) 1–10. https://doi.org/10.1186/1471-2156-14-12.

[10] P. Vikram, B.P.M. Swamy, S. Dixit, R. Singh, B.P. Singh, B. Miro, A. Kohli, A. Henry, N.K. Singh, A. Kumar, Drought susceptibility of modern rice varieties: An effect of linkage of drought tolerance with undesirable traits, Sci. Rep. (2015). https://doi.org/10.1038/srep14799.

[11] A. Kumar, N. Sandhu, S. Dixit, S. Yadav, B.P.M. Swamy, N.A.A. Shamsudin, Marker-assisted selection strategy to pyramid two or more QTLs for quantitative trait-grain yield under drought, Rice. (2018). https://doi.org/10.1186/s12284-018-0227-0.

[12] M.S. BP, H.U. Ahmed, A. Henry, R. Mauleon, S. Dixit, P. Vikram, R. Tilatto, S.B. Verulkar, P. Perraju, N.P. Mandal, Genetic, physiological, and gene expression analyses reveal that multiple QTL enhance yield of rice mega-variety IR64 under drought, PLoS One. 8 (2013) e62795.

[13] L. Ge, H. Chen, J.F. Jiang, Y. Zhao, M.L. Xu, Y.Y. Xu, K.H. Tan, Z.H. Xu, K. Chong, Overexpression of OsRAA1 causes pleiotropic phenotypes in transgenic rice plants, including altered leaf, flower, and root development and root response to gravity, Plant Physiol. (2004). https://doi.org/10.1104/pp.104.041996.

[14] Y. Yamamoto, N. Kamiya, Y. Morinaka, M. Matsuoka, T. Sazuka, Auxin biosynthesis by the YUCCA genes in rice, Plant Physiol. (2007). https://doi.org/10.1104/pp.106.091561.

[15] Q. Zhang, J. Li, W. Zhang, S. Yan, R. Wang, J. Zhao, Y. Li, Z. Qi, Z. Sun, Z. Zhu, The putative auxin efflux carrier OsPIN3t is involved in the drought stress response and drought tolerance, Plant J. 72 (2012) 805–816.

[16] H. Bian, Y. Xie, F. Guo, N. Han, S. Ma, Z. Zeng, J. Wang, Y. Yang, M. Zhu, Distinctive expression patterns and roles of the miRNA393/TIR1 homolog module in regulating flag leaf inclination and primary and crown root growth in rice (Oryza sativa), New Phytol. 196 (2012) 149–161. https://doi.org/10.1111/j.1469-8137.2012.04248.x.

[17] T. Yoshikawa, M. Ito, T. Sumikura, A. Nakayama, T. Nishimura, H. Kitano, I. Yamaguchi, T. Koshiba, K. Hibara, Y. Nagato, The rice FISH BONE gene encodes a tryptophan aminotransferase, which affects pleiotropic auxin-related processes, Plant J. 78 (2014) 927–936.

[18] Y. Zhao, S. Cheng, Y. Song, Y. Huang, S. Zhou, X. Liu, D.-X. Zhou, The interaction between rice ERF3 and WOX11 promotes crown root development by regulating gene expression involved in cytokinin signaling, Plant Cell. 27 (2015) 2469–2483.

[19] M. Xu, L. Zhu, H. Shou, P. Wu, A PIN1 family gene, OsPIN1, involved in auxin-dependent adventitious root emergence and tillering in rice, Plant Cell Physiol. (2005). https://doi.org/10.1093/pcp/pci183.

[20] Y. Zhao, Y. Hu, M. Dai, L. Huang, D.X. Zhou, The WUSCHEL-Related homeobox gene WOX11 is required to activate shoot-borne crown root development in rice, Plant Cell. (2009). https://doi.org/10.1105/tpc.108.061655.

[21] X.F. Wang, F.F. He, X.X. Ma, C.Z. Mao, C. Hodgman, C.G. Lu, P. Wu, OsCAND1 is required for crown root emergence in rice, Mol. Plant. (2011). https://doi.org/10.1093/mp/ssq068.

[22] Y. Inukai, T. Sakamoto, M. Ueguchi-Tanaka, Y. Shibata, K. Gomi, I. Umemura, Y. Hasegawa, M. Ashikari, H. Kitano, M. Matsuoka, Crown rootless1, which is essential for crown root formation in rice, is a target of an Auxin Response Factor in auxin signaling, Plant Cell. (2005). https://doi.org/10.1105/tpc.105.030981.

[23] H. Liu, S. Wang, X. Yu, J. Yu, X. He, S. Zhang, H. Shou, P. Wu, ARL1, a LOB-domain protein required for adventitious root formation in rice, Plant J. (2005). https://doi.org/10.1111/j.1365-313X.2005.02434.x.

[24] Y.M. Woo, H.J. Park, M. Su'udi, J. Il Yang, J.J. Park, K. Back, Y.M. Park, G. An, Constitutively wilted 1, a member of the rice YUCCA gene family, is required for maintaining water homeostasis and an appropriate root to shoot ratio, Plant Mol. Biol. (2007). https://doi.org/10.1007/s11103-007-9203-6.

[25] Y. Kitomi, A. Ogawa, H. Kitano, Y. Inukai, CRL4 regulates crown root formation through auxin transport in rice, Plant Root. 2 (2008) 19–28. https://doi.org/10.3117/plantroot.2.19.

[26] S. Liu, J. Wang, L. Wang, X. Wang, Y. Xue, P. Wu, H. Shou, Adventitious root formation in rice requires OsGNOM1 and is mediated by the OsPINs family, Cell Res. 19 (2009) 1110–1119.

[27] Y. Kitomi, H. Ito, T. Hobo, K. Aya, H. Kitano, Y. Inukai, The auxin responsive AP2/ERF transcription factor CROWN ROOTLESS5 is involved in crown root initiation in rice through the induction of OsRR1, a type-A response regulator of cytokinin signaling, Plant J. 67 (2011) 472–484. https://doi.org/10.1111/j.1365-313X.2011.04610.x.

[28] J. Yuan, D. Chen, Y. Ren, X. Zhang, J. Zhao, Characteristic and expression analysis of a metallothionein gene, OsMT2b, down-regulated by cytokinin suggests functions in root development and seed embryo germination of rice, Plant Physiol. (2008). https://doi.org/10.1104/pp.107.110304.

[29] Y.H. Chen, Y.Y. Chao, Y.Y. Hsu, C.Y. Hong, C.H. Kao, Heme oxygenase is involved in nitric oxide- and auxin-induced lateral root formation in rice, Plant Cell Rep. (2012). https://doi.org/10.1007/s00299-012-1228-x.

[30] Y. Wang, D. Wang, T. Gan, L. Liu, W. Long, Y. Wang, M. Niu, X. Li, M. Zheng, L. Jiang, J. Wan, CRL6, a member of the CHD protein family, is required for crown root development in rice, Plant Physiol. Biochem. 105 (2016) 185–194. https://doi.org/10.1016/j.plaphy.2016.04.022.

[31] A. Nakamura, I. Umemura, K. Gomi, Y. Hasegawa, H. Kitano, T. Sazuka, M. Matsuoka, Production and characterization of auxin-insensitive rice by overexpression of a mutagenized rice IAA protein, Plant J. 46 (2006) 297–306. https://doi.org/10.1111/j.1365-313X.2006.02693.x.

[32] Y. Morita, J. Kyozuka, Characterization of OsPID, the rice ortholog of PINOID, and its possible involvement in the control of polar auxin transport, Plant Cell Physiol. 48 (2007) 540–549. https://doi.org/10.1093/pcp/pcm024.

[33] H. Zhao, T. Ma, X. Wang, Y. Deng, H. Ma, R. Zhang, J. Zhao, OsAUX1 controls lateral root initiation in rice (Oryza sativaL.), Plant Cell Environ. (2015). https://doi.org/10.1111/pce.12467.

[34] B. Kang, Z. Zhang, L. Wang, L. Zheng, W. Mao, M. Li, Y. Wu, P. Wu, X. Mo, OsCYP2, a chaperone involved in degradation of auxin-responsive proteins, plays crucial roles in rice lateral root initiation, Plant J. 74 (2013) 86–97. https://doi.org/10.1111/tpj.12106.

[35] Z.X. Zhu, Y. Liu, S.J. Liu, C.Z. Mao, Y.R. Wu, P. Wu, A gain-of-function mutation in OsIAA11 affects lateral root development in rice, Mol. Plant. 5 (2012) 154–161. https://doi.org/10.1093/mp/ssr074.

[36] Y. Kitomi, H. Inahashi, H. Takehisa, Y. Sato, Y. Inukai, OsIAA13-mediated auxin signaling is involved in lateral root initiation in rice, Plant Sci. 190 (2012) 116–122. https://doi.org/10.1016/j.plantsci.2012.04.005.

[37] X. Chen, J. Shi, X. Hao, H. Liu, J. Shi, Y. Wu, Z. Wu, M. Chen, P. Wu, C. Mao, OsORC3 is required for lateral root development in rice, Plant J. 74 (2013)

339–350. https://doi.org/10.1111/tpj.12126.

[38] S.H. Cho, S.C. Yoo, H. Zhang, D. Pandeya, H.J. Koh, J.Y. Hwang, G.T. Kim, N.C. Paek, The rice narrow leaf2 and narrow leaf3 loci encode WUSCHEL-related homeobox 3A (OsWOX3A) and function in leaf, spikelet, tiller and lateral root development, New Phytol. (2013). https://doi.org/10.1111/nph.12231.

[39] Y. Uga, E. Hanzawa, S. Nagai, K. Sasaki, M. Yano, T. Sato, Identification of qSOR1, a major rice QTL involved in soil-surface rooting in paddy fields, Theor. Appl. Genet. (2012). https://doi.org/10.1007/s00122-011-1688-3.

[40] J. Huang, C.M. Kim, Y.H. Xuan, J. Liu, T.H. Kim, B.K. Kim, C.D. Han, Formin homology 1 (OsFH1) regulates root-hair elongation in rice (Oryza sativa), Planta. 237 (2013) 1227–1239. https://doi.org/10.1007/s00425-013-1838-8.

[41] C. Wang, S. Li, S. Ng, B. Zhang, Y. Zhou, J. Whelan, P. Wu, H. Shou, Mutation in xyloglucan 6-xylosytransferase results in abnormal root hair development in Oryza sativa, J. Exp. Bot. (2014). https://doi.org/10.1093/jxb/eru189.

[42] W. Ding, Z. Yu, Y. Tong, W. Huang, H. Chen, P. Wu, A transcription factor with a bHLH domain regulates root hair development in rice, Cell Res. (2009). https://doi.org/10.1038/cr.2009.109.

[43] Z. Yu, B. Kang, X. He, S. Lv, Y. Bai, W. Ding, M. Chen, H.T. Cho, P. Wu, Root hair-specific expansins modulate root hair elongation in rice, Plant J. (2011). https://doi.org/10.1111/j.1365-313X.2011.04533.x.

[44] T. Yuo, M. Toyota, M. Ichii, S. Taketa, Molecular cloning of a root hairless gene rth1 in rice, Breed. Sci. 59 (2009) 13–20. https://doi.org/10.1270/jsbbs.59.13.

[45] J. Huang, C.M. Kim, Y.H. Xuan, S.J. Park, H.L. Piao, B. Il Je, J. Liu, T.H. Kim, B.K. Kim, C.D. Han, OsSNDP1, a Sec14-nodulin domain-containing protein, plays a critical role in root hair elongation in rice, Plant Mol. Biol. 82 (2013) 39–50. https://doi.org/10.1007/s11103-013-0033-4.

[46] M.K. Chul, H.P. Sung, I.J. Byoung, H.P. Su, J.P. Soon, L.P. Hai, Y.E. Moo, L. Dolan, C.D. Han, OsCSLD1, a cellulose synthase-like D1 gene, is required for root hair morphogenesis in rice, Plant Physiol. (2007). https://doi.org/10.1104/pp.106.091546.

[47] T. Yuo, K. Shiotani, N. Shitsukawa, A. Miyao, H. Hirochika, M. Ichii, S. Taketa, Root hairless 2 (RTH2) mutant represents a loss-of-function allele of the cellulose synthase-like gene OSCSLD1 in rice (Oryza sativa L.), Breed. Sci. (2011). https://doi.org/10.1270/jsbbs.61.225.

[48] N. Kamiya, H. Nagasaki, A. Morikami, Y. Sato, M. Matsuoka, Isolation and characterization of a rice WUSCHEL-type homeobox gene that is specifically expressed in the central cells of a quiescent center in the root apical meristem, Plant J. 35 (2003) 429–441. https://doi.org/10.1046/j.1365-313X.2003.01816.x.

[49] S. Sun, M. Gu, Y. Cao, X. Huang, X. Zhang, P. Ai, J. Zhao, X. Fan, G. Xu, A constitutive expressed phosphate transporter, OsPht1;1, modulates phosphate uptake and translocation in phosphate-replete rice, Plant Physiol. (2012). https://doi.org/10.1104/pp.112.196345.

[50] J. Ni, G. Wang, Z. Zhu, H. Zhang, Y. Wu, P. Wu, OsIAA23-mediated auxin signaling defines postembryonic maintenance of QC in rice, Plant J. (2011). https://doi.org/10.1111/j.1365-313X.2011.04698.x.

[51] H. Cui, M.P. Levesque, T. Vernoux, J.W. Jung, A.J. Paquette, K.L. Gallagher, J.Y. Wang, I. Blilou, B. Scheres, P.N. Benfey, An evolutionarily conserved

mechanism delimiting SHR movement defines a single layer of endodermis in plants, Science (80-.). 316 (2007) 421–425. https://doi.org/10.1126/science.1139531.

[52] N. Kamiya, J. Itoh, A. Morikami, Y. Nagato, M. Matsuoka, The SCARECROW gene's role in asymmetric cell divisions in rice plants, Plant J. 36 (2003) 45–54.

[53] H. Jia, H. Ren, M. Gu, J. Zhao, S. Sun, X. Zhang, J. Chen, P. Wu, G. Xu, The phosphate transporter gene ospht1;8 is involved in phosphate homeostasis in rice, Plant Physiol. (2011). https://doi.org/10.1104/pp.111.175240.

[54] S. Ray, J. Vijayan, R.K. Sarkar, Germination stage oxygen deficiency (GSOD): An emerging stress in the era of changing trends in climate and rice cultivation practice, Front. Plant Sci. 7 (2016) 1–4. https://doi.org/10.3389/fpls.2016.00671.

[55] Jaiswal, 基因的改变NIH Public Access, Bone. 23 (2014) 1–7. https://doi.org/10.1111/tpj.12497.OsMOGS.

[56] L. Jia, B. Zhang, C. Mao, J. Li, Y. Wu, P. Wu, Z. Wu, OsCYT-INV1 for alkaline/neutral invertase is involved in root cell development and reproductivity in rice (Oryza sativa L.), Planta. 228 (2008) 51–59. https://doi.org/10.1007/s00425-008-0718-0.

[57] Y. Qi, S. Wang, C. Shen, S. Zhang, Y. Chen, Y. Xu, Y. Liu, Y. Wu, D. Jiang, OsARF12, a transcription activator on auxin response gene, regulates root elongation and affects iron accumulation in rice (Oryza sativa), New Phytol. 193 (2012) 109–120.

[58] Y. Inukai, T. Sakamoto, Y. Morinaka, M. Miwa, M. Kojima, E. Tanimoto, H. Yamamoto, K. Sato, Y. Katayama, M. Matsuoka, H. Kitano, ROOT GROWTH INHIBITING, a Rice Endo-1,4-β-d-Glucanase, Regulates Cell Wall Loosening and is Essential for Root Elongation, J. Plant Growth Regul. (2012). https://doi.org/10.1007/s00344-011-9247-3.

[59] J.W. Zhang, L. Xu, Y.R. Wu, X.A. Chen, Y. Liu, S.H. Zhu, W.N. Ding, P. Wu, K.K. Yi, OsGLU3, a putative membrane-bound endo-1,4-beta-glucanase, is required for root cell elongation and division in rice (Oryza sativa L.), Mol. Plant. (2012). https://doi.org/10.1093/mp/ssr084.

[60] Y. Zou, X. Liu, Q. Wang, Y. Chen, C. Liu, Y. Qiu, W. Zhang, OsRPK1, a novel leucine-rich repeat receptor-like kinase, negatively regulates polar auxin transport and root development in rice, Biochim. Biophys. Acta - Gen. Subj. 1840 (2014) 1676–1685. https://doi.org/10.1016/j.bbagen.2014.01.003.

[61] C. Qin, Y. Li, J. Gan, W. Wang, H. Zhang, Y. Liu, P. Wu, OsDGL1, a homolog of an oligosaccharyltransferase complex subunit, is involved in N-glycosylation and root development in rice, Plant Cell Physiol. 54 (2013) 129–137. https://doi.org/10.1093/pcp/pcs159.

[62] H. Jiang, S. Wang, L. Dang, S. Wang, H. Chen, Y. Wu, X. Jiang, P. Wu, A novel short-root gene encodes a glucosamine-6-phosphate acetyltransferase required for maintaining normal root cell shape in rice, Plant Physiol. 138 (2005) 232–242. https://doi.org/10.1104/pp.104.058248.

[63] Y. Uga, K. Sugimoto, S. Ogawa, J. Rane, M. Ishitani, N. Hara, Y. Kitomi, Y. Inukai, K. Ono, N. Kanno, H. Inoue, H. Takehisa, R. Motoyama, Y. Nagamura, J. Wu, T. Matsumoto, T. Takai, K. Okuno, M. Yano, Control of root system architecture by DEEPER ROOTING 1 increases rice yield under drought conditions, Nat. Genet. (2013). https://doi.org/10.1038/ng.2725.

[64] H.M. Seo, Y. Jung, S. Song, Y. Kim, T. Kwon, D.H. Kim, S.J. Jeung, Y.B. Yi, G. Yi, M.H. Nam, J. Nam, Increased expression of OsPT1, a high-affinity phosphate transporter, enhances phosphate acquisition in rice, Biotechnol. Lett. (2008). https://doi.org/10.1007/s10529-008-9757-7.

[65] M. Yan, X. Fan, H. Feng, A.J. Miller, Q. Shen, G. Xu, Rice OsNAR2.1 interacts with OsNRT2.1, OsNRT2.2 and OsNRT2.3a nitrate transporters to provide

uptake over high and low concentration ranges, Plant, Cell Environ. 34 (2011) 1360–1372. https://doi.org/10.1111/j.1365-3040.2011.02335.x.

[66] S. Qiu, N. Ma, S. Che, Y. Wang, X. Peng, G. Zhang, G. Wang, J. Huang, Repression of OsEXPA3 expression leads to root system growth suppression in rice, Crop Sci. (2014). https://doi.org/10.2135/cropsci2013.11.0746.

[67] S.A. Ramesh, R. Shin, D.J. Eide, D.P. Schachtman, Differential metal selectivity and gene expression of two zinc transporters from rice, Plant Physiol. (2003). https://doi.org/10.1104/pp.103.026815.

[68] Y. Zhang, L. Tan, Z. Zhu, L. Yuan, D. Xie, C. Sun, TOND1 confers tolerance to nitrogen deficiency in rice, Plant J. 81 (2015) 367–376. https://doi.org/10.1111/tpj.12736.
